# Role of mitochondrial DNA in diabetes Mellitus Type I and Type II

**DOI:** 10.1016/j.sjbs.2022.103434

**Published:** 2022-09-11

**Authors:** Bandar Ali Al-Ghamdi, Jawhra M. Al-Shamrani, Ahmed M. El-Shehawi, Intisar Al-Johani, Bandar G. Al-Otaibi

**Affiliations:** a Department of Cardiology and Cardiac Surgery, King Fahad Armed Forces Hospital, Jeddah, Saudi Arabia; b Department of Biotechnology, Taif University, Taif City, Saudi Arabia

**Keywords:** Diabetes mellitus, Diabetes Mellitus Type I, Diabetes Mellitus Type II, Inflammation in diabetes, Maternally inherited diabetic syndrome, The role of mitochondria in diabetes, The search for diabetes, mtDNA mutations in diabetes patients, Diabetes-A

## Abstract

Morbidity and mortality from diabetes mellitus and associated illnesses is a major problem across the globe. Anti-diabetic medicines must be improved despite existing breakthroughs in treatment approaches. Diabetes has been linked to mitochondrial dysfunction. As a result, particular mitochondrial diabetes kinds like MIDD (maternally inherited diabetes & deafness) and DAD (diabetic autonomic dysfunction) have been identified and studied (diabetes and Deafness). Some mutations as in mitochondrial DNA (mtDNA), that encodes for a significant portion of mitochondrial proteins as well as mitochondrial tRNA essential for mitochondrial protein biosynthesis, are responsible for hereditary mitochondrial diseases. Tissue-specificity and heteroplasmy have a role in the harmful phenotype of mtDNA mutations, making it difficult to generalise findings from one study to another. There are a huge increase in the number for mtDNA mutations related with human illnesses that have been identified using current sequencing technologies. In this study, we make a list on mtDNA mutations linked with diseases and diabetic illnesses and explore the methods by which they contribute to the pathology's emergence.

## 1 Introduction

Deterioration in the capacity of cells to reproduce and maintain homeostasis is a hallmark of ageing, which affects all bodily systems. Even in the absence of usual inflammation triggers, age-related immune system dysregulation leads to the generation of pro-inflammatory cytokines. An “inflammation” of this age-related low-level systemic inflammation has been identified in several human illnesses (Flynn et al., 2019). In addition to the primary cytokine markers (IL-1, IL-6, IL-8, IL-13 & IL-18, IL-1 receptor (IL-1RN), tumour necrosis factor (TNF), ifn (IFN)-, IFN-, and tumour growth factor (TGF)-), previous investigations have found a variety of molecular markers indicating a pro-inflammatory state. TNFRSF members 1A and 1B, serum amyloid A, and C-reactive protein (CRP) have recently been added to the list (Ferrucci and Fabbri, 2018). Human illnesses such as cardiovascular disease, cancer, diabetes, mental illness, and neurodegeneration are all known to be exacerbated by inflammation. Inflammaging-related processes have also been linked to a reduced lifespan and early mortality (Fabbri et al., 2015, Stepanova et al., 2015).

Cell metabolism and cell development and proliferation are dependent on the proper functioning of mitochondria. The mitochondrial malfunction has been linked to a wide variety of human illnesses and age-related conditions, including cancer and diabetes, cardiovascular disease, and atherosclerosis. Proper mitochondrial turnover is necessary for a cell to maintain a functioning mitochondrial population that reacts to changes in energy demand. Mitophagy, a special form of autophagy, may be used to break down faulty or superfluous mitochondria into smaller pieces, which can then be eliminated via mitophagy. Mitochondrial fusion may be used to combine functional pieces into a new organelle. Mitophagy is a vital process for maintaining a healthy mitochondrial population, and its lack may play a significant role in a variety of illnesses (Aryaman et al., 2019).

As a key regulator of cell differentiation and death, mitophagy has a critical function to play in cell biology. Furthermore, it seems to have a significant impact on the immunological response. PINK1-Parkin-dependent mitophagy and PINK1-Parkin-independent mitophagy have been found. Parkin (an E3 ubiquitin ligase essential for PINK1 protein degradation) is found in the cytoplasm and is responsible for the degradation of this protein under normal circumstances. Although mitochondrial PINK1 is autophosphorylated in response to cellular injury or stress, Parkin is attracted to the mitochondria, and mitophagy is stimulated when mitophagy is activated. The fact that Parkin can still migrate to the mitochondria in PINK1-deficient animals and initiate mitophagy suggests that PINK1 deficiency may have alternate compensatory mechanisms (Onishi et al., 2021). Reactive oxygen species (ROS) generation is one of the most common symptoms linked with mitophagy malfunction and mitochondrial dysfunction (Zuo et al., 2019). The effectiveness of mitochondrial activity declines with age but is often linked to age-related diseases, according to converging lines of study (Kauppila et al., 2017).

Mutations in the mitochondrial DNA (mtDNA) accumulate over time, resulting in decreased energy production and an increase in reactive oxygen species (ROS) formation. Cells may create additional mitochondria in response to mild oxidative stress to produce more energy. The number of mitochondria is controlled by a process known as mitochondrial biogenesis, which involves a number of transcription factors and regulators. Varied organs exhibit different mitochondrial biogenesis, which reflects the various needs of each organ's unique inherent function (Wang et al., 2009). Further mutagenesis is caused by ROS damage to mtDNA. Defective copies of mitochondrial DNA (mtDNA) might accumulate over time, raising the overall heteroplasmy level of dangerous mutations, since mitochondrial DNA repair processes are not as dependable as nuclear ones. As a result, the course of the illness is sped up. Diabetes and its consequences have been linked to mitochondrial dysfunction, oxidative stress, and inflammation in previous studies (Baltrusch, 2016, Oguntibeju, 2019). We summaries the most recent findings in the understanding of the function of mitochondria mutations in the development and progression of diabetes in this paper.

## 2 Inflammation in diabetes

When blood glucose levels remain high over an extended period, it is known as diabetes. Diabetes problems affecting the cardiovascular system, kidneys, eyes, and neurological system are all linked to untreated or poorly controlled diabetes. Diabetes already affects 8.8 percent of the world's population (422 million), and this figure is expected to rise over time. Nearly-nine in ten (89 %) of people with diabetes are diagnosed with type 2 diabetes (T2D). The prevalence of type 1 diabetes (T1D) is around 9 % globally. When the beta cells in the pancreas are destroyed by an autoimmune assault, it results in type 1 diabetes (T1D). Regular insulin injections are the mainstay of care for people with type 2 diabetes. Insulin deficit may also occur in later stages of T2D if cells fail to respond properly to insulin signals.

Healthy living, food compliance, calorie restriction, and regular exercise are all recommended methods to prevent the onset of type 2 diabetes (T2D) (International Diabetes Federation, 2019). Diabetes during pregnancy (also known as gestational diabetes mellitus or GDM) affects around six percent of pregnancies but is curable in 90 % of those instances once the baby is born. GDM patients, on the other hand, are more likely to acquire type 2 diabetes later on in life (Institute, 2021). When the m.A3243G mtDNA point mutation affects the gene for tRNA-Leu, it results in mitochondrial diabetes (MIDD or DAD). Intestinal malabsorption, cardiomyopathy, or renal failure are all signs of this condition, which is inherited from the mother.

### 2.1 Type 1 diabetes

As an autoimmune illness, Type 1 Diabetes (T1D) may be diagnosed. *T*-cells of the organism target just the pancreatic -cells in this scenario. These two types of *T*-cells, CD8, and CD4 play a major role in the killing of beta-cells in specific Antigens recognized by these immune cells including many -cell antigens, including proinsulin and proinsulin, insulinoma antigen, IGRP, GAD65, as well as islet amyloid polypeptide (Gonzalez-Duque et al., 2018). The precise pathways that lead to this autoimmune disease are still a mystery. Autoantigen recognition doesn't occur in healthy persons, although autoreactive *T*-cells are present (Bender et al., 2020). Antigen presentation may be altered and autoreactive *T*-cell recruitment to -cells influenced by microenvironmental variables such as cytokines, oxidative stress, and chemokines (Yeo et al., 2020). Cytokine activation is required for normal pancreatic - and -cell function. Stress alters cytokine production in both healthy individuals and those with type 1 diabetes (Rajendran et al., 2020). Inflammation-related interleukin (IL)-1b is produced by -cells, whereas IL-6 is produced by both - and -cells, which helps regulate glucose homeostasis (Rajendran et al., 2020, Anquetil et al., 2017).

Some additional cytokines are involved in the pathophysiology of T1D and the immunological assault on insulin-producing beta cells, including interferon-gamma, CXCL10, IL-6, IL-17, IL-21, tumour necrosis factor (TNF), and others (Lu et al., 2020). Multiple studies have demonstrated the important function of MHC class I in the development of T1D. In the absence of MHCI, autoreactive T cells were unable to return to islets (Whitener et al., 2017). The upregulation of MHCI by IFN- and MHCII by IFN- has been shown, respectively, in the literature (Marroqui et al., 2017, Scott et al., 2018).

It was clear from these findings that inflammation played an important role in the onset of T1D. CXCL9, CXCL10, and CCL5 cytokines and MHCI were overexpressed in apoptotic -cells exposed to pro-inflammatory cytokines, resulting in endoplasmic reticulum (ER) distress and apoptosis. Pro-inflammatory cytokines have been linked to a decrease in insulin process enzyme (PC1/3, PC2, and CPE) and a disruption in glucose-mediated insulin release, leading to the buildup of proinsulin in -cells. There is evidence that mitochondria play a major role in cell death caused by cytokines because they limit pyruvate utilization, increase superoxide levels, and decrease ATP production (Barlow et al., 2018).

### 2.2 Type 2 diabetes

Hyperglycemia is a hallmark of Type 2 diabetes and may lead to a wide range of health problems, including cardiovascular disease, renal disease, and vision loss. Ultimately it can lead to death. As obesity-induced insulin resistance and Type 2 Diabetes (T2D) develop, low-level chronic inflammation is a major contributor. Increased apoptosis of -cells, increased cytokine production, and impaired insulin biosynthesis are all associated with T2D pancreatic chronic inflammation, which occurs in conjunction with hyperglycemia with fatty infiltration (Horii et al., 2020). Adipose tissue is a major source of pro-inflammatory cytokines, such as TNF, MCP-1, and IL-6, in both people and animals, according to some studies. T2D may be caused by a combination of fat and inflammation (Crujeiras et al., 2019, 2019,, He et al., 2021).

Insulin resistance has been linked to the production of the pro-inflammatory cytokines described above. This action might be either direct or indirect, depending on how macrophages infiltrate and apoptosis of -cells is promoted. In addition, a general mechanism for hypothalamic dysfunction has been characterised, which involves IKK- and NF-B activation (Zhang et al., 2013). Neuronal injury has been linked to cognitive deterioration in patients with type 2 diabetes (T2D) (Bury et al., 2021, Dyer et al., 2020). Furthermore, age-related mitochondrial dysfunction has long been linked to insulin resistance (Bhansali et al., 2017).

### 2.3 Maternally inherited diabetic syndrome

When a mutation in the mitochondrial tRNA(Leu)(UUR) gene causes both maternally inherited diabetes and deafness, the condition is known as Maternally Inherited Diabetes and Deafness (MIDD). Neurosensory deafness is a common complication of multiple sclerosis. In contrast to other forms of diabetes, the pathophysiology of MIDD is linked directly to a mutation in the mitochondrial DNA that is passed down via the maternal line. The tRNA(Leu)(UUR) gene is affected by the mitochondrial mutation m.A3243G. Since this mutation affects all mitochondrial protein production as well as the stability of several mitochondrial proteins, it has a very wide-ranging effect.

The MELAS syndrome (mitochondrial myopathy, encephalopathy, lactic acidosis, and stroke-like episodes) is also known to be caused by the m.A3243G mutation, which is linked to diabetes. The diverse effects of the m.A3243G mutation on various organs and tissues may account for the observed phenotypic variation. m.A3243G mutation in the pancreas is linked to decreased insulin and glucagon production in the beta and cells, respectively (Norose et al., 2020). It's fairly uncommon for MIDD to not be linked to insulin resistance, thus insulin therapy is not necessary (McMillan et al., 2019). The disease pathogenesis is likely based on the failure of -cells to react to glucose stimulation promptly. Research on m.A3243G and its many physiologic manifestations has been detailed elsewhere (Cañadas-Garre et al., 2019) after its discovery.

## 3 The role of mitochondria in diabetes

### 3.1 Mitochondrial genome

**Fig. 1:**
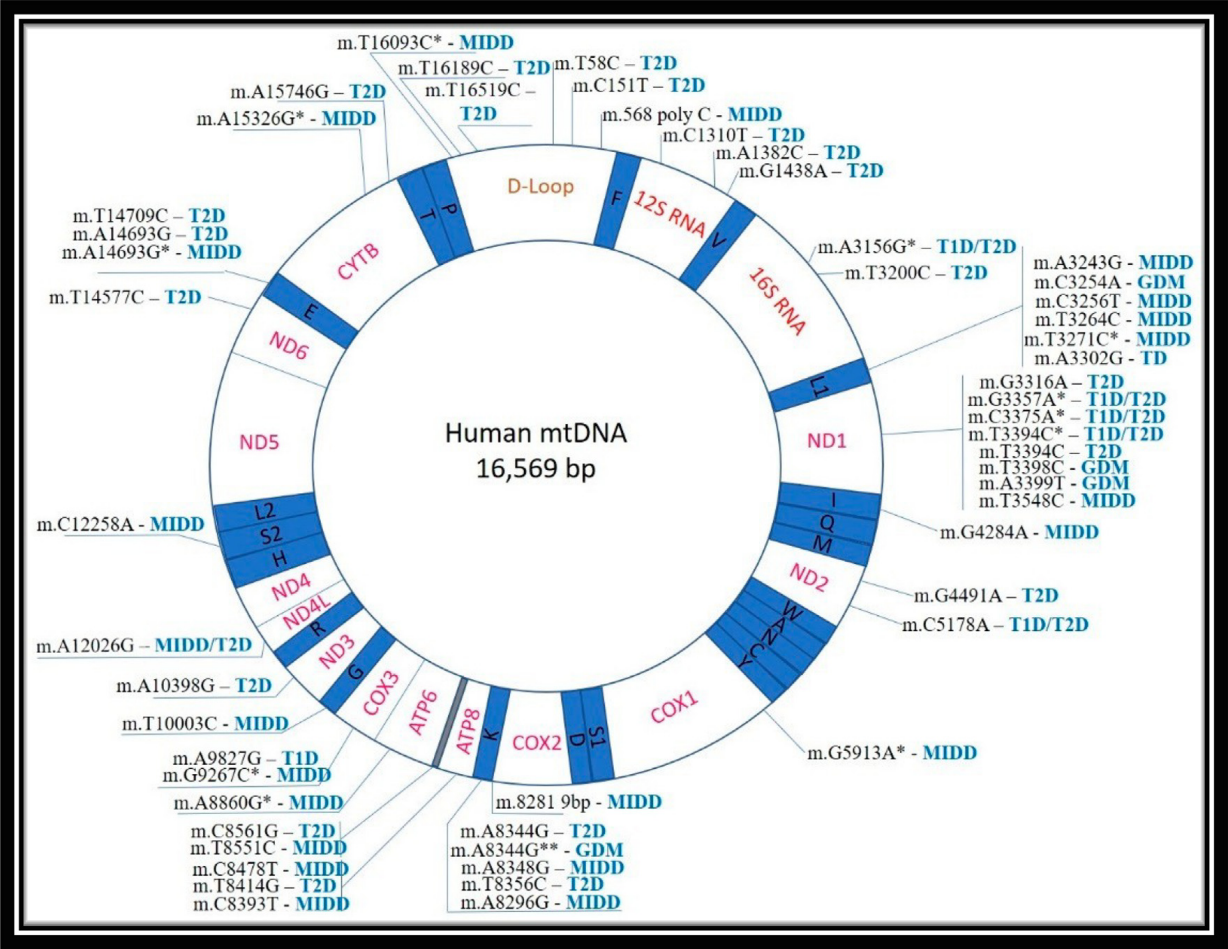
Map of diabetes-related mtDNA mutations. *- a new mutation has been discovered in additional to m.3243G.

As a result of their endosymbiosis with proteobacteria, mitochondria are thought to maintain several bacterial properties such as their genomic structure. This is consistent with the current theory. Human mtDNA is a circular molecule of 16,569 bp in length. For example, the heavy (H) and light (L) nucleotide compositions are different: the H one is more guanine-rich, whereas L is more of the latter. The mtDNA encodes and synthesises a significant portion (but not all) of mitochondrial proteins, making mitochondria largely independent. The 13 mtDNA-encoded proteins that makeup four of the five primary enzymatic reactions involved in oxidative phosphorylation (complexes I, III-IV) were critical. In addition, the mitochondrial DNA (mtDNA) encodes two ribonucleic acids (rRNA) genes (the small 12S and big 16S rRNAs) and 22 transfer RNA (tRNA) genes (Fig. 1). While, animal mtDN have qualities that make it a perfect genetic marker as shown in the early investigation. Animal mtDNA is typically a circular, compact molecule that is 17 Kb in size, with little changes in size. It has 22 tRNA genes, 2 rRNA genes, and 13 protein-coding genes. Double uni-parental inheritance is a well-known and idiomorphic example of paternal transmission in animals (DUI). Females give their mtDNA to both female and male offspring in this unusual manner of transmission, whereas males transmit their mtDNA only to male offspring. The challenging nature of altering mitochondrial genome is primarily responsible for comparative scarcity of animal models of mtDNA mutation (Dunn et al., 2012, Zhang et al., 1993).

The mtDNA coding region is tightly packed. For the whole mtDNA, the single non-coding region (d-loop) performs a regulatory function, including H-strand transcription promoters and replication start sites (Boguszewska and Szewczuk, 2020). The fact that practically every mutation has the potential to induce functional problems and even disease because of the way the mitochondrial genome is organised is understandable. In different tissues and organs, as well as under different physiological conditions, a cell's mtDNA copy number might fluctuate.

Homoplasmic mutations are those that affect all copies of mtDNA, whereas heteroplasmic mutations affect just a subset of mtDNA copies. Mutations in the mitochondrial DNA (mtDNA) may have a variety of molecular and cellular consequences depending on the degree of heteroplasmy. When a particular amount of mutation heteroplasmy is attained, a certain phenotype is often seen (Aryaman et al., 2019). Functional wild-type copies of mtDNA may hide mutations. To modify phenotypes, a high amount of heteroplasmy is required (often more than 70 %), and this heteroplasmy may be tissue-specific (Stewart and Chinnery, 2015).

### 3.2 The search for diabetes-related mitochondrial mutations

Currently, this oral glucose tolerance testing is the main and most straightforward method of diagnosing diabetes. As a result of the test having identical cut-off values for all kinds of diabetes, this method is unable to make a distinction between them. Since metabolic alterations in cells are linked to diabetes, it's reasonable to assume that mitochondrial malfunction contributes to the disease. However, the causal link between mtDNA mutations and diabetes-related illnesses is difficult to demonstrate save from a few maternally inherited diseases. These investigations are complicated by the fact that pathological symptoms are reliant on the heteroplasmy levels, that mtDNA mutations accumulate and develop with age, and that the consequences of mtDNA mutations vary in various organs and tissues (Karaa and Goldstein, 2015, Wang et al., 2021).

The role of mitochondrial function connected with mitochondrial DNA in the diagnosis of diabetes has been actively investigated. Some of the most plausible causes of diabetes have been identified and the processes by which they work have been suggested. Insulin transmission, production and secretion, and insulin responsiveness in the peripheral muscle are all known to be altered with mitochondrial dysfunction in diabetes. Symptoms and patient features (body mass index, excessive urination, polydipsia, the dynamics of weight gain/loss, the existence of auto-antibodies as well as ketones, and levels of C-peptide) have a role in the control of diabetes mellitus and insulin therapy.

A reasonable T2D medication should be prescribed if autoantibodies and C-peptide levels are negative and there are no evident symptoms (Zhang et al., 2021). Genetic testing should be considered in specific situations. The existence of mtDNA mutations should be examined if the maternal transmission or sex symptoms are suspected of being present. These include WES, mitoexome, WGS and mtDNA sequencing for mtDNA molecular analysis (Cortez et al., 2020). For the most part, the focus of WES is on finding specific coding variations in a particular genome. Nuclear mutations, in particular, may benefit from this technique. There is room for improvement in terms of speed and accuracy (Wysham and Shubrook, 2020).

The ability to identify somatic & mitochondrial mutations using WES has been increased by focusing on the mitochondrial exome or a restricted number of genes (Mustafa et al., 2020). It is possible to identify mtDNA mutations using WGS, which is a more sophisticated method, and it has significant benefits in time and cost (Di Resta et al., 2018, Han et al., 2017). NGS relies on one strand of DNA for sequencing purposes, while duplex sequencing employs the use of both DNA strands.

Using a single-reaction primer-free amplification, MitoRS was recently developed. While this approach is robust and sensitive, the low amount of heteroplasmy makes it well-suited for big samples (Schon et al., 2020). Samples for genetic analysis should be collected and analysed with care. It is required to examine a variety of tissues and organs since the heteroplasmy levels vary from one tissue or organ to the next. Muscle, for example, has a higher than average heteroplasmy frequency for mtDNA mutations (Ahn et al., 2015). The degree of mtDNA mutation heteroplasmy varies substantially across people. Additional mtDNA mutations can not only be prenatally inherited but may also develop sporadically, as was revealed in monozygotic twins with diabetes (Marquis et al., 2017). For the D-loop of a mitochondrial genome, the MAF and heteroplasmy levels were found to be considerably greater in liver samples. Heteroplasmic mutation rates were influenced by age and positive selection, according to the study (Guo et al., 2019).

Data like this lend credence to the “survival of the slowest” argument first put out in 1997 (Avital et al., 2012, Hübner et al., 2019) D-loop mutations in mitochondria have been shown to limit respiration and minimise ROS generation, according to this notion. ROS damages and eliminates mitochondria that are devoid of altered mtDNA, increasing the proportion of mutated mtDNA in cells. Non-synonymous heteroplasmies, on the other hand, are targeted by selection for hereditary mutations. Analysis of mtDNA mutations, natural polymorphism, and heteroplasmy has been done before for various neurodegenerative disorders and some cancers (De Grey et al., 1997), but it is still a subject for further inquiry for diabetes mellitus.

### 3.3 Analysis of pathogenic mtDNA mutations in diabetes patients

Until recently, “mtDNA disorders” were regarded as rare ailments with severe symptoms (heart and circulatory system, neurodegenerative & age-related diseases and syndromes, and multisystem consequences) connected with mtDNA mutations. Polymorphisms in the mtDNA sequence were once thought to be homoplasmic, whereas harmful mutations became heteroplasmic (Floros et al., 2018).

It was the development of a cybrid (cytoplasmic hybrid) cells technique in 1992 that led to its widespread usage in examining cellular abnormalities, particularly in the context of the MELAS syndrome. Certain clinical features associated with diabetes must be present for the use of cybrid cells to detect mutations in the mitochondrial DNA. It's ideal for examining mutations in a small number of people. More sensitive and high-throughput approaches are required for larger populations and low-frequency mtDNA mutations. In addition, cybrid technology may be used to investigate the putative pathogenic processes of mtDNA mutations. A few mtDNA mutations have been linked to diabetes, most notably MIDD and MELAS, which contain m.A3243G alterations in their mtDNA sequences.

The causal involvement in disease development has yet to be confirmed for several additional mutations, though (see Table S1). There have been 54 mtDNA mutations linked to various types of diabetes so far, and more are expected to be discovered (Fig. 1). There are a large number of genetic mutations that are linked to type 2 diabetes and type 2 diabetes mellitus (28 and 23, respectively). Only a few genetic alterations are linked to T1D and GDM (6 and 4, respectively). The mitochondrial genome has several hotspot locations where diabetes-related mutations are most common. Six mutations are linked to T2D and MIDD in the D-loop region, a non-coding portion of the mtDNA molecule that controls replication and translation (Fig. 1) (Stewart and Chinnery, 2021).

Different types of cancer have been linked to mutations in this particular region of the mitochondrial DNA molecule because of its high degree of variability. However, further research is needed to determine exactly how D-loop mutations lead to the diabetes phenotype. The increased mtDNA replication, as well as mtDNA depletion caused by these mutations, is expected to have an impact on mitochondrial function as a whole. Cells and tissues that are most susceptible to disease development are affected by the ATP deficit. mtDNA depletion has been found in some diseases including cancer in humans. When it comes to T2D, mtDNA depletion has been linked to decreased insulin production and an increase in mutations in the mitochondrial DNA (mtDNA) (Wallace, 1992).

Different kinds of diabetes are connected with many mtDNA abnormalities in the mitochondrial translational machinery (rRNA and tRNA genes). Mutations related to T2D, MIDD, or GDM are found in the L1 and K areas (Fig. 1). Other human disorders, such as ocular myopathy, myoclonic epilepsy, ragged red muscle fibers (MERRF), and cardiomyopathies, have been linked to mutations in these locations. Comparison between the hotspot of diabetes-related mitochondrial illness mtDNA mutations with other mitochondrial disorders is particularly intriguing.

In cells that rely significantly on energy generation, the impacts of these abnormalities naturally disrupt mitochondrial protein synthesis. This results in general mitochondrial dysfunction, decreased ATP production, and cellular loss of function. Mutations linked to diabetes may also impair mitochondrial protein-coding genes. There have been seven mutations in the ND1 gene, which codes for NADH–ubiquinone oxidase subunit 1, and many others in the genes encoding other subunits that have been linked to various forms of diabetes (Fig. 1). The cellular redox equilibrium may be disturbed as a result of these alterations. T2D diabetic individuals with ND1 and ND2 gene mutations were found to have impaired ATP production in leukocytes (Chomyn et al., 1992). Mutations in the mitochondrial DNA (mtDNA) linked to a wide range of human disorders have been shown to occur most often in regions critical to the replication, translation, or synthesis of mitochondrial proteins.

Diabetes-related mutations and mitochondriopathies, which have impaired energy metabolism, share a large number of mutations. Because of this overlap, mtDNA mutations may be linked to disease through shared pathophysiological pathways. For each mutation, however, a causal link must be shown by employing techniques such as cybrid or animal models to verify it. Age-related phenotypic changes and the development of mtDNA mutations may be linked or reliant on a shared external source, such as an increase in oxidative or exposure to specific toxins.

## 4 Study of molecular mechanisms of Diabetes-Associated mtDNA mutations

### 4.1 Mouse models

We learned a lot about how mitochondrial abnormalities cause diabetes by studying mice. The NOD/Lt mouse strain is more susceptible to T1D than the ALR/Lt mouse strain (Cree et al., 2008). SNPs in the mt-Nd2 (MtA4738C) & mt-Co3 (MtA9827G) loci, which are associated with the development of T1D, may be identified by the crossing of these two strains. To protect from free radicals in the body, maternally passed on ALR/Lt-specific SNPs in the mt-Nd2 and mt-Co3 genes look beneficial (Jiang et al., 2021). Mitophagy, as shown in p53-deficient mice cells, plays a critical role in the development of diabetes. Mitophagy in -cells is reduced by p53′s direct interaction with Parkin, which inhibits insulin production in high-glucose circumstances (Mathews et al., 2003). The involvement of p53 in pancreatic -cells mitophagy dysregulation offers additional evidence linking cancer, inflammation, and diabetes, in addition to its function as a tumour suppressor but also a controller of redox or glucose metabolism (Mathews et al., 2005). Newly found gene expression regulators – small non-coding mini RNAs (miRNAs) may also affect the expression of mitochondrial genes.

Mitochondrial miRNAs are shown to be abnormal in diabetic animals, which contributes to our knowledge of the disease causes and suggests a new treatment approach. For the research and therapy of diabetic cardiaomyopathy, animal studies have found mitochondrial miRNA that induces mitochondrial target gene expression (Hoshino et al., 2014). The C57BL/6J-mtFVB/N (B6-mtFVB) mice strain has a naturally occurring m.G7778T polymorphism (AspTyr substitution) in the MT-ATP8 gene. High ROS production, decreased glucose sensitivity, and decreased insulin secretion were all seen in pancreatic -cells with these mutations (Aning and Cheok, 2019).

Researchers found that this mitochondrial alteration was linked to an increased risk of autoimmune disorders (such as Type 1 diabetes, pancreatitis, and nephritis), as well as decreased female fertility. According to the study, abnormally shaped mitochondria produced more ROS and ATP than normal mitochondria (Li et al., 2020). Mutation (m.5172, in the OriL) was found recently in a mouse strain (AKR/J (B6-mtAKR) on a C57BL/6J (B6) background. Most people's mtDNA has 60–70 % “11A” at the 5172 positions, although studies have shown that “12A” heteroplasmy is associated with a shorter lifespan, decreased mtDNA copy number, as well as worse glucose and lipid metabolic function. Interestingly, the heteroplasmy level did not affect the OXPHOS function (Weiss et al., 2012). The MT-ND6 gene's pathogenic m.G13997A mutation was examined in an elderly mito-mice ND6M mouse strain to see its impact. Hyperglycaemia and B-lymphoma were more common in those who had the m.G13997A mutation, which increases ROS generation. There is evidence that mitochondrial mutations and respiratory abnormalities are connected to the evolution of diabetes and heart disease (Yu et al., 2009).

### 4.2 Preclinical and clinical studies

Mechanisms for the development of type 2 diabetes should be explored on pancreatic beta cells, which are critical for disease pathogenesis. Because -cell glucose absorption is insulin-independent, the glucose concentration that -cells can metabolise is determined by the amount of plasma glucose. Through the Ca2+-dependent channel, processed glucose controls insulin release by determining the cell's ATP/ADP ratio. Mutations in the mitochondrial DNA (mtDNA), which affect protein synthesis and the creation of ATP, have a negative impact on insulin synthesis, glucose absorption, and signaling.

Toxic chemicals were also shown to disrupt normal Ca2+-dependent signaling and to be related to hyperglycemia in mitochondria affected by the disease (Hirose et al., 2018). Respiratory chain activity has been linked to glucose-dependent insulin production in early investigations (Hashizume et al., 2012). Recent studies corroborate these findings and show that mtDNA is vulnerable to ROS-mediated oxidation caused by the respiratory chain (Sha et al., 2020). Many chemicals have been examined preclinically and are now being tested in clinical trials for the treatment of mitochondrial diseases. However, no effective treatments exist. As a general rule, therapies depend on the replenishing of an antioxidant pool and the correction of metabolic pathways that have been impacted by secondary metabolites to sustain respiratory chain operations (Malaisse et al., 1979). Lifestyle and regular exercise and training have proved to be more effective than pharmaceuticals in improving respiratory chain efficiency, overall physiological and mitochondrial function.

When drugs were paired with a tailored diet (balanced, low-calorie, ketogenic) (Bist et al., 2016), greater results might be attained. Research has demonstrated that a ketogenic diet has antioxidant and anti-inflammatory qualities as well as a positive effect on muscle health as well as mitochondrial biogenesis (Almannai et al., 2020). Mitochondrial mutations yielded the most promising outcomes. In vitro studies have shown that a ketogenic diet may selectively eliminate heteroplasmic mtDNA mutations, raise total mtDNA levels, and improve tissue health (Bottani et al., 2020, Ma and Suzuki, 2019). Despite several encouraging results, additional research is needed into the dietary strategy in the therapy of mitochondrial-related disorders.

### 4.3 Pharmacotherapy

Diabetic patients with mitochondrial abnormalities are not currently receiving specific pharmacological therapy (Emperador et al., 2019). Individualized therapy is necessary due to the wide range of mtDNA mutations that influence distinct elements of mitochondrial function. It has been shown that Metformin, the most frequent medication for T2D, may produce lactic acidosis in patients with mitochondrial diabetes. At least ten T2D-associated variations have been found in nuclear DNA by genome-wide association analyses, which also highlighted the participation of numerous variants with small effects. Despite mtDNA's differences from nuclear DNA, the gene products of the mitochondrial and nuclear genomes interact and are expressed in a coordinated manner. It is unclear how faulty oxidative metabolism relates to mitochondrial dysfunction and requires more research (Filograna et al., 2019, Parikh et al., 2017).

When metformin is used, it inhibits mitochondrial glycerophosphate dehydrogenase within the liver, causing lactic acidosis. However, lactic acidosis is more often linked with underlying conditions and illnesses of the heart, liver, and kidney. With considerable care and frequent lactate monitoring, metformin may be an option in certain situations (Keidai et al., 2019, Madiraju et al., 2014). SGLT-2i, GLP-1 RA similar components, and their derivatives are the most effective and extensively used therapies for T2D. Blood sugar and body weight drop, heart failure, and non-fatal myocardial infarction are all improved as well as inflammation, liver steatosis as well as uric acid concentrations, thanks to the sodiumglucose carrier protein 2 (SGLT-2) inhibition in the intestinal mucosa. Analogs of GLP-1, or incretin mimetics, are used to treat T2D and obesity largely within the pancreas and liver. Reduced appetite and decreased stroke risk are two of the side effects of this medication (Yeung et al., 2018). Cardiomyopathy, atherosclerosis, and hypertension may all be caused by mitochondrial abnormalities that increase ROS generation, making them highly harmful to the cardiovascular system.

Mitochondrial abnormalities in diabetic people have been linked to cardiovascular disease. As a result, SGLT-2i, GLP-1 RA, and other cardioprotective compounds provide considerable advantages over existing therapies (Bonora et al., 2020). SGLT-2i (Empagliflozin) does have a direct effect on mitochondrial functioning, biogenesis, and membrane potential, as shown in a rat model. SGL-2i may operate within mitochondria by Tfam and NRF-1 signalling pathways (Dogruel and Balci, 2019). Increases in Mfn1 and Mfn2 and Fis1 protein levels were shown to enhance mitochondrial functioning by limiting membrane depolarization, lowering oxidative stress damage, and enhancing the ADP/ATP ratio (Zheng et al., 2018).

This might be even more advantageous for older patients because of the known age-related deregulation of SGLT-2 owing to the decreased equilibrium between both the mitochondria with sarcoplasmic reticulum Ca2+ (Shao et al., 2019). GLP-1 RAs, on the other hand, have been shown to exert their effects directly on the mitochondria. Liraglutide has been demonstrated to diminish chronic inflammation by reducing TNF and IL-1 in the hippocampus, as well as indicators of mitochondria stresses (Durak et al., 2018) and promoting the synthesis of anti-inflammatory proteins (arginase 1, IL-10, and TGF) (Olgar et al., 2020) using a rat model. Parkin-mediated mitophagy and reduced oxidative stress damage have been shown to have cardioprotective properties of liraglutide (Wang et al., 2018). Some GLP1 RAs may be hazardous because of their systemic effects, particularly in older individuals with concomitant conditions. Hypoglycemia and weight loss may both be exacerbated by a reduced calorie intake and a diminished appetite. Both diarrhea and vomiting are frequent symptoms of gastroenteritis (Diz-Chaves et al., 2018, Qiao et al., 2018).

At this point, it is well accepted that inflammation and age-related declines in mitochondrial efficiency are partly caused by the accumulation of mtDNA mutations. However, the precise pathophysiology whereby the mtDNA mutation led to the creation of diabetes and accompanying comorbidities has not yet been investigated in depth. Pancreatic beta cells' high susceptibility to mitochondrial oxidative seems to be the most likely cause of insulin production failure. Other additional possibilities might be responsible, including hyperglycaemia and alterations in lipid metabolism as well as Ca2+ signaling violations and other yet-to-be-determined causes (Bettge et al., 2017, Rayner et al., 2021).

## 5 Conclusions

It is not uncommon for diabetics to have an altered mtDNA. The vast majority of mtDNA are unique to the group they affect, although some of them are more common than others. Researchers and clinicians face the most difficult challenge when attempting to detect and model heteroplasmic mtDNA mutations in vitro. Many mitochondrial diabetes-related mutations have been found in mtDNA regions responsible for the replication of mitochondrial DNA, translational machinery, or specific mitochondrial genes that encode important proteins, such as NADH–ubiquinone oxidation subunits. As a result of these mutations, the mtDNA cell growth and as a whole mitochondrial function may be affected, reducing power generation or increasing ROS generation.

Certain cell types, including pancreatic -cells, may be particularly vulnerable to these processes. By evaluating the genomes of family and friends, it is now possible to determine the possibility of disease development in future generations by analysing population-specific as well as unique mtDNA mutations and to begin treatment and disease prevention interventions as soon as necessary. We can better understand the impact of mutations on metabolism if we investigate each genome one at a time. As a result, we could take another step forward toward medical technology and effective treatment for every patient in the future.

## Declaration of Competing Interest

The authors declare that they have no known competing financial interests or personal relationships that could have appeared to influence the work reported in this paper.
